# Supplementary material for: Microbial Metabolic Limitations and Their Relationships with Sediment Organic Carbon Across Lake Salinity Gradient in Tibetan Plateau
Source: Microorganisms. 2025 Mar 11;13(3):629. doi: 10.3390/microorganisms13030629 (PMC11945249; doi:10.3390/microorganisms13030629)
Supplement: Supplementary file 1 [file microorganisms-13-00629-s001.zip › microorganisms-3460879-supplementary.pdf]

Supporting information for *Microorganisms* article:

# Microbial Metabolic Limitations and Their Relationships with Sediment Organic Carbon Across Lake Salinity Gradient in Tibetan Plateau

**Weizhen Zhang [1,2,*], Jianjun Wang [3,4], Yun Li [3], Chao Song [5], Yongqiang Zhou [3], Xianqiang Meng [3] and Ruirui Chen [6]**

[1] Center for Pan-Third Pole Environment, Lanzhou University, Lanzhou 730000, China
[2] Chayu Monsoon Corridor Observation and Research Station for Multi-Sphere Changes, Xizang Autonomous Region, Chayu 860600, China
[3] State Key Laboratory of Lake Science and Environment, Nanjing Institute of Geography and Limnology, Chi-nese Academy of Sciences, Nanjing 210008, China; jjwang@niglas.ac.cn (J.W.); liyun@niglas.ac.cn (Y.L.); yqzhou@niglas.ac.cn (Y.Z.); xqmeng@niglas.ac.cn (X.M.)
[4] University of Chinese Academy of Sciences, Beijing 100049, China
[5] State Key Laboratory of Herbage Improvement and Grassland Agro-Ecosystems, College of Ecology, Lanzhou University, Lanzhou 730000, China; chaosong@lzu.edu.cn
[6] College of Chemical Engineering, Nanjing Forestry University, Nanjing 210008, China; rrchen@njfu.edu.cn
* Correspondence: zhangwz@lzu.edu.cn

Contents:

Table S1. Description of environmental variables and extracellular enzyme activities.
Table S2. Comparations of nutrients and extracellular enzyme characteristics between lakes in this and other studies.
Figure S1. Comparations of environmental factors among 4 salinity levels.
Figure S2. Linear regressions between water salinity and extracellular enzyme characteristics.
Figure S3. Stoichiometry of the relative proportion of enzymatic C:N versus C:P in different lakes.
Figure S4. The rarefaction curve plotted after rarefying the denoised bacterial and fungal sequences.
Figure S5. Stoichiometry of the relative proportion of enzymatic C:(C+N) versus C:(C+P) for lakes in other studies.
Figure S6. Stacked bars showing bacterial and fungal community compositions.
Figure S7. Linear regressions of sediment pH with enzyme activities and microbial metabolic limitations.

**Table S1.** Description of environmental variables and extracellular enzyme activities.

| | | min | max | median | mean |
|---|---|---|---|---|---|
| **Climate factors** | Mean annual temperature (MAT, ºC) | -3.6 | 2.6 | -0.8 | -0.8 |
| | Mean annual precipitation (MAP, mm) | 5.8 | 33.4 | 22.4 | 22.1 |
| **Geographical factor** | Elevation (m) | 4031 | 5008 | 4542 | 4518 |
| **Water physiochemical features** | Depth (m) | 3.8 | 55.7 | 18.2 | 21.4 |
| | Temperature (ºC) | 11.9 | 18.9 | 15.0 | 15.1 |
| | Salinity (‰) | 0.13 | 31.06 | 1.24 | 4.66 |
| | Chlorophyll-*a* (Chl-*a*, μg $L^{-1}$) | 0.10 | 10.50 | 0.44 | 1.33 |
| | Total nitrogen (TN, mg $L^{-1}$) | 0.14 | 3.38 | 0.85 | 1.05 |
| | Total phosphorus (TP, mg $L^{-1}$) | 0.005 | 3.829 | 0.036 | 0.368 |
| | TN:TP (molar ratio) | 1.03 | 238.96 | 42.72 | 56.81 |
| **Sediment physiochemical features** | pH | 7.11 | 10.06 | 8.77 | 8.74 |
| | Conductivity (μs $cm^{-1}$) | 317.0 | 7760.0 | 640.5 | 1565.8 |
| | Total organic carbon (TOC, g $kg^{-1}$) | 1.8 | 152.6 | 28.5 | 42.7 |
| | Total nitrogen (TN, g $kg^{-1}$) | 0.32 | 19.8 | 3.91 | 4.81 |
| | Total phosphorus (TP, g $kg^{-1}$) | 0.19 | 1.69 | 0.54 | 0.56 |
| | TN:TP (molar ratio) | 2.53 | 226.23 | 16.62 | 28.44 |
| | TOC:TN (molar ratio) | 6.06 | 22.71 | 9.08 | 10.18 |
| | TOC:TP (molar ratio) | 16.59 | 2033.53 | 137.73 | 289.77 |
| | Dissolved organic carbon (DOC, g $kg^{-1}$) | 0.1 | 7.4 | 0.7 | 1.2 |
| | $PO_4^{3-}$ (mg $kg^{-1}$) | 0.39 | 35.42 | 1.84 | 4.15 |
| | $NO_3^-$ (mg $kg^{-1}$) | 0.03 | 4.81 | 0.49 | 0.95 |
| | $NO_2^-$ (mg $kg^{-1}$) | 0.14 | 1.23 | 0.36 | 0.41 |
| | $NH_4^+$ (mg $kg^{-1}$) | 21.98 | 134.42 | 34.86 | 45.86 |
| **Extracellular enzyme activities** | β-glucosidase (BG, nmol MUF·$g^{-1}$·$h^{-1}$) | 20.50 | 569.45 | 108.76 | 144.69 |
| | Cellobiohydrolase (CBH, nmol MUF·$g^{-1}$·$h^{-1}$) | 0.00 | 124.63 | 17.24 | 25.52 |
| | β-N-acetylglucosaminidase (NAG, nmol MUF·$g^{-1}$·$h^{-1}$) | 0.34 | 361.67 | 46.56 | 75.59 |
| | Leucine amino peptidase (LAP, nmol AMC·$g^{-1}$·$h^{-1}$) | 0.00 | 86.18 | 10.82 | 17.55 |
| | Alkaline phosphatase (AP, nmol MUF·$g^{-1}$·$h^{-1}$) | 0.00 | 152.13 | 22.17 | 34.01 |

**Table S2**. Comparations of the mean and range values of water depth, nutrient contents, extracellular enzyme activity and ecoenzymatic stoichiometry vector features of Lakes in this study with those in other saline lakes like Qinghai Lake and Hulun Lake in the northern semi-arid area, and freshwater lakes including Fuxian Lake in the southwestern Yunnan-Guizhou Pleatau and 38 shallow lakes along the Yangtze-Huaihe River basin of China. Detailed information for the abbreviation of variables is listed in Table S1. Only means with different letters between lakes are significantly different ($p \leq 0.05$).

| | Qinghai Lake (Sep, 2020) ※ | Hulun Lake (Jun, 2020) ⁋ | Fuxian Lake (Jul, 2019) ‡ | Lakes along Yangtze-Huaihe River basin (Aug, 2019) § | Lakes in Tibetan Plateau (July, 2018; Aug~Sep, 2020) † |
|---|---|---|---|---|---|
| **n (site No.)** | 27 | 19 | 46 | 80 | 44 |
| **Water depth (m)** | 25.2 (14.3-29.7) [ab] | 5.7 (3.8-6.4) [bc] | 68.4 (2.0-155.0) [ad] | 2.3 (0.3-6.4) [e] | 21.4 (0.5-55.7) [acd] |
| **Water TP ($mg\ L^{-1}$)** | 0.04 (0.01-0.09) [a] | 0.13 (0.07-0.17) [b] | 0.06 (0.01-0.27) [a] | 0.11 (0.02-0.62) [b] | 0.368 (0.005-3.829) [a] |
| **Water TN ($mg\ L^{-1}$)** | 1.04 (0.29-2.80) [a] | 1.12 (0.81-1.35) [a] | 0.45 (0.24-1.54) [b] | 1.22 (0.28-3.56) [a] | 1.05 (0.14-3.38) [a] |
| **Water TN:TP (molar ratio)** | 64.54 (30.31-199.18) [a] | 20.42 (16.95-25.46) [b] | 23.44 (9.85-54.31) [b] | 34.96 (5.82-87.55) [c] | 56.81 (1.03-238.96) [c] |
| **Sediment TP ($g\ kg^{-1}$)** | 0.62 (0.50-0.73) [ac] | 0.80 (0.18-1.10) [bc] | 1.89 (0.50-5.09) [d] | 0.84 (0.38-1.87) [b] | 0.56 (0.19-1.69) [a] |
| **Sediment TC ($g\ kg^{-1}$)** | 65.25 (47.54-82.05) [a] | 35.43 (2.32-56.80) [b] | 49.35 (9.94-104.07) [c] | - | - |
| **Sediment TN ($g\ kg^{-1}$)** | 2.89 (1.51-4.19) [ab] | 2.06 (0.20-3.40) [b] | 3.14 (0.76-5.55) [ab] | - | 4.81 (0.32-19.8) [a] |
| **Sediment TN:TP (molar ratio)** | 10.19 (6.08-13.45) [a] | 4.77 (0.50-11.20) [b] | 5.14 (2.19-7.52) [b] | - | 28.44 (2.53-226.23) [a] |
| **BG** | 458.12 (82.83-1149.47) [a] | 76.80 (0-260.26) [bc] | 89.04 (8.92-296.62) [bc] | 65.46 (0-325.50) [b] | 144.69 (20.5-569.45) [cd] |
| **CBH** | 159.11 | 7.00 | 2.36 | 13.64 | 25.52 |

| | | | | | |
|---|---|---|---|---|---|
| | (27.60-644.08) [a] | (0-15.80) [bc] | (0.00-30.33) [b] | (0-122.48) [cd] | (0-124.63) [cd] |
| **NAG** | 114.38<br>(47.70-258.22) [ac] | 45.50<br>(0-132.02) [be] | 74.03<br>(5.87-181.45) [ce] | 39.23<br>(0-196.86) [bd] | 75.59<br>(0.34-361.67) [be] |
| **LAP** | 92.69<br>(28.82-230.96) [a] | 16.90<br>(5.86-51.37) [b] | 87.50<br>(12.46-164.73) [a] | 52.71<br>(4.47-309.95) [a] | 17.55<br>(0-86.18) [a] |
| **AP** | 24.90<br>(0-52.52) [b] | 10.00<br>(0-172.35) [a] | 35.79<br>(0-373.12) [bc] | 22.68<br>(0-251.39) [d] | 34.01<br>(0-152.13) [a] |
| **Proportion of C-acquiring enzyme activity (%)** | 67<br>(38-91) [a] | 46<br>(0-93) [bd] | 31<br>(7-71) [c] | 41<br>(0-75) [b] | 67<br>(49-86) [ad] |
| **Proportion of N-acquiring enzyme activity (%)** | 21<br>(4-46) [a] | 32<br>(0-73) [bc] | 34<br>(7-72) [bc] | 29<br>(0-63) [ac] | 31<br>(12-51) [ab] |
| **Proportion of P-acquiring enzyme activity (%)** | 13<br>(3-33) [a] | 21<br>(2-100) [a] | 35<br>(11-80) [b] | 30<br>(3-100) [bc] | 1<br>(0-6) [a] |
| **Vector length (microbial C limitation)** | 1.125<br>(0.793-1.348) [a] | 0.933<br>(0-1.366) [a] | 0.678<br>(0.259-1.175) [b] | 0.835<br>(0.496-1.209) [c] | 1.08<br>(0.51-1.31) [a] |
| **Vector angle (microbial N/P limitation)** | 42.07<br>(29.66-48.92) [bc] | 33.15<br>(0-56.77) [b] | 45.47<br>(18.94-77.62) [ac] | 45.06<br>(20.95-64.46) [ac] | 39.40<br>(27.58-53.72) [bd] |

※23 sites covering the whole Lake Qinghai, the largest saline lake in China, were sampled in our previous study [1].
⁋19 sites covering the whole Lake Hulun, the largest lake in northern China, were sampled in our previous study [2].
‡, §Unpublished data. There were 1 to 4 sampling sites for each lake along Yangtze-Huaihe River basin.
†This study.

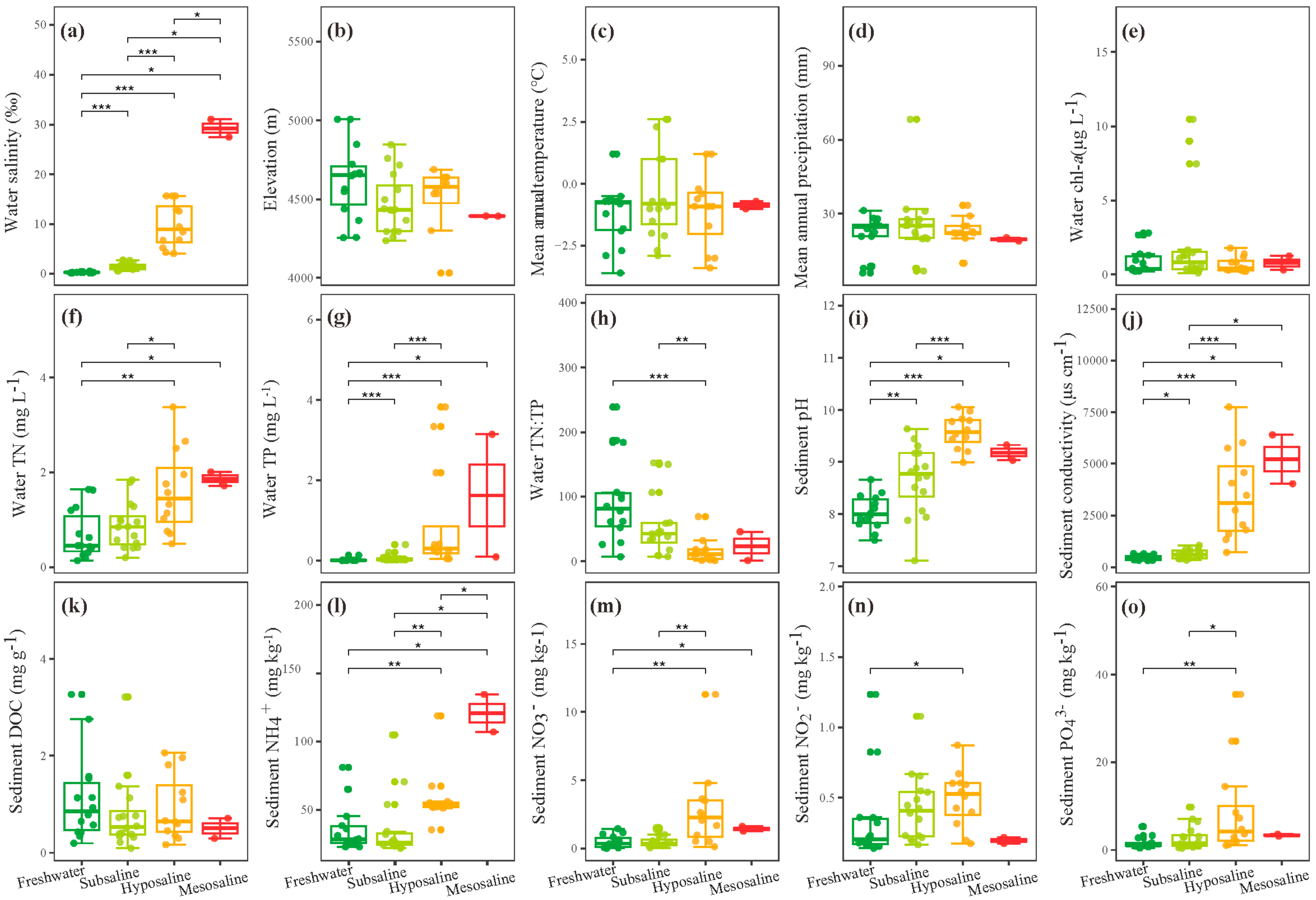


**Figure S1.** Box plots comparing physiochemical (a, e-o), geographical (b) and climatic (c-d) features between different salinity regimes. The asterisks above the boxes indicate the significance of the pairwise difference based on the multiple comparisons using least significant difference (LSD) for One-way analyses of variance. $^{*}$ $p < 0.05$, $^{**}$ $p < 0.01$, $^{***}$ $p < 0.001$.

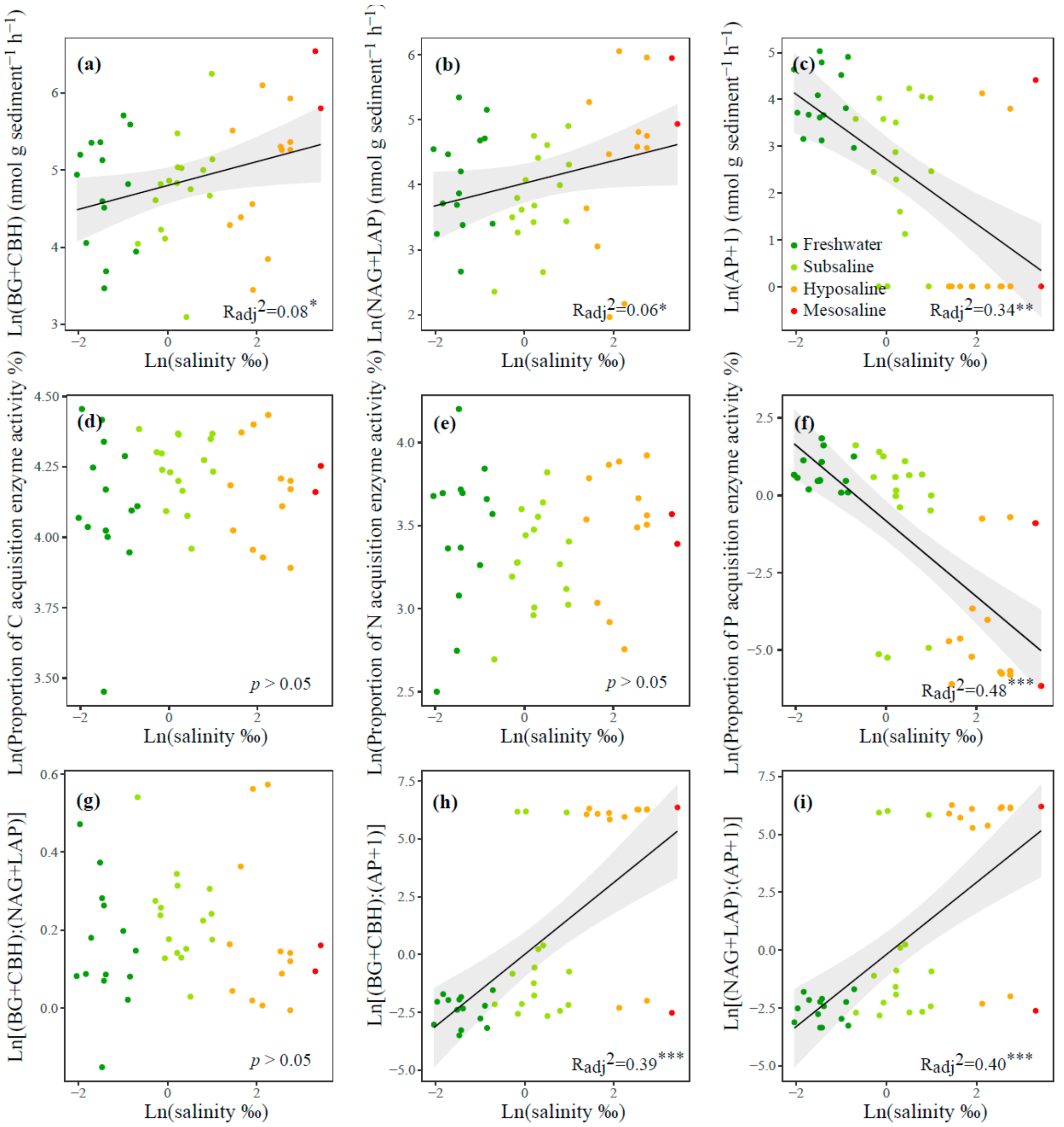


**Figure S2.** Linear regressions of water salinity with enzyme activities involved in C-cycle (BG+CBH) (a), N-cycle (NAG+LAP) (b) and P-cycle (AP) (c), with proportions of C, N and P acquisition enzymes activities to total activities (d-f), and with the ratio of C- to N-acquiring, C- to P-acquiring and N- to P-acquiring enzymatic activities (g-i). The horizonal and vertical axes are log-transformed from original data to facilitate the fittings (a-c, g-i). Only the significant fitted linear regressions are plotted with 95% confidence intervals filled in gray. The adjusted $R^2$ values of the linear models are denoted. ** $p < 0.01$, *** $p < 0.001$. BG, β-1,4-glucosidase; CBH, β-D-cellobiosidase; NAG, β-1,4-N-acetylglucosaminidase; LAP, L-leucine aminopeptidase; AP, alkaline phosphatase.

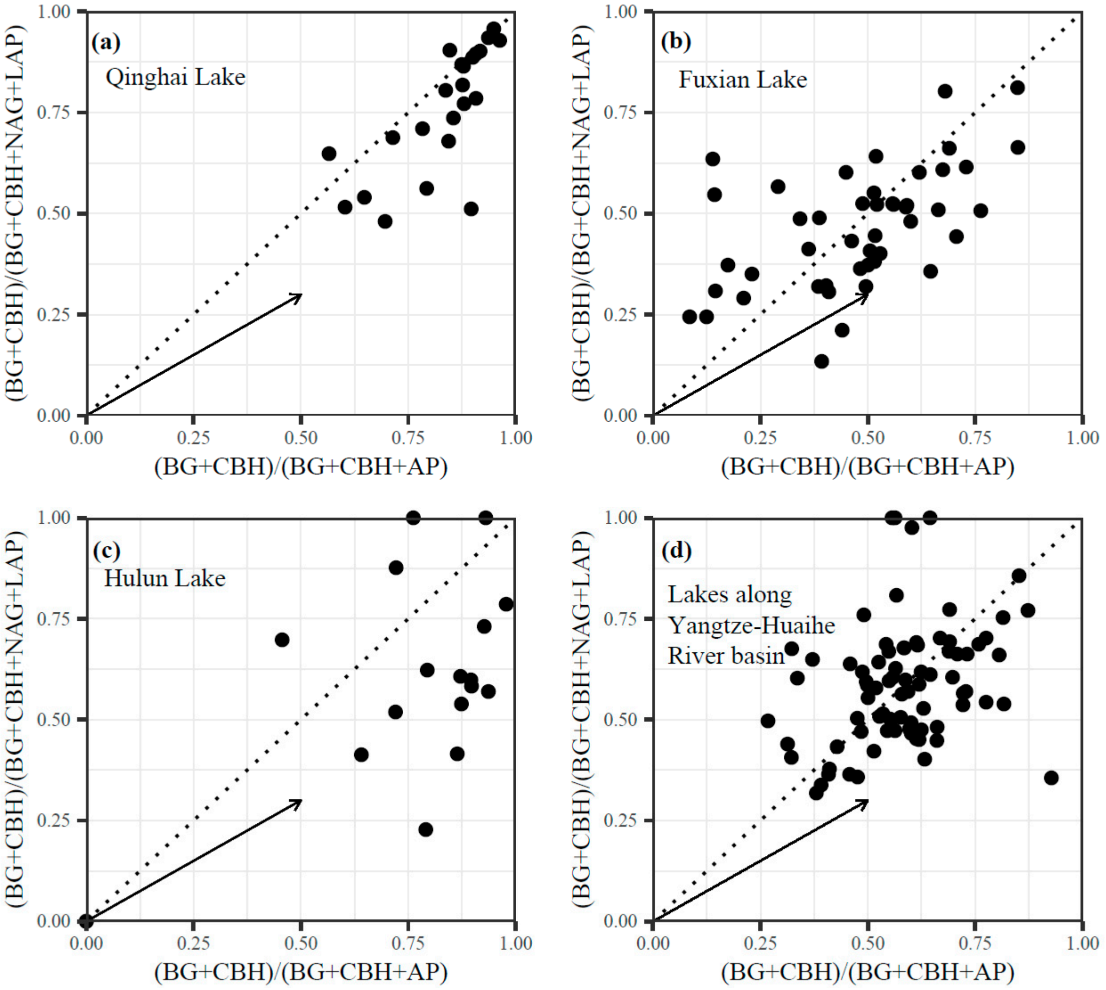


**Figure S3.** Stoichiometry of the relative proportion of enzymatic C:(C+N) versus C:(C+P) in different lakes including Qinghai Lake (a), Fuxian Lake (b), Hulun Lake (c) and lakes along Yangtze-Huaihe River basin (d).

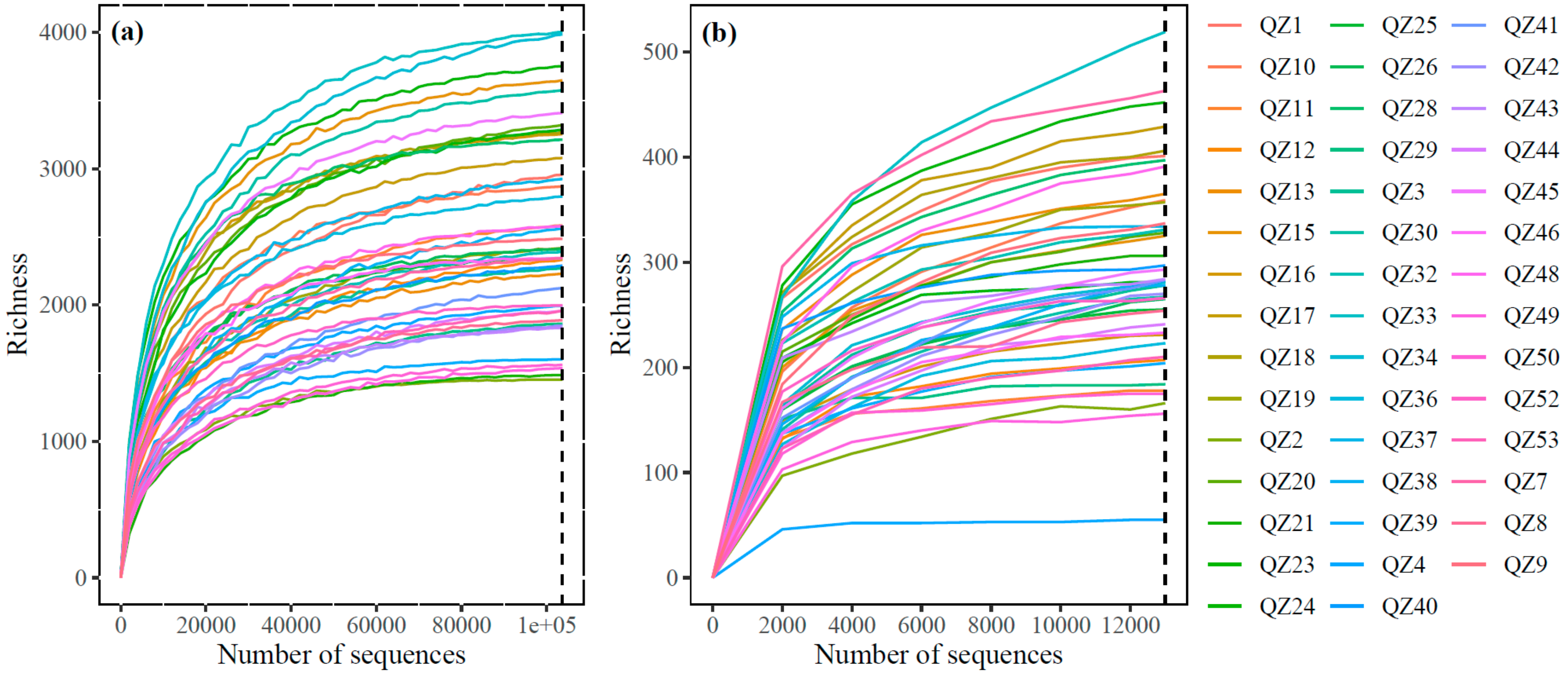


**Figure S4.** The rarefaction curve plotted after rarefying the denoised bacterial (a) and fungal (b) sequences.

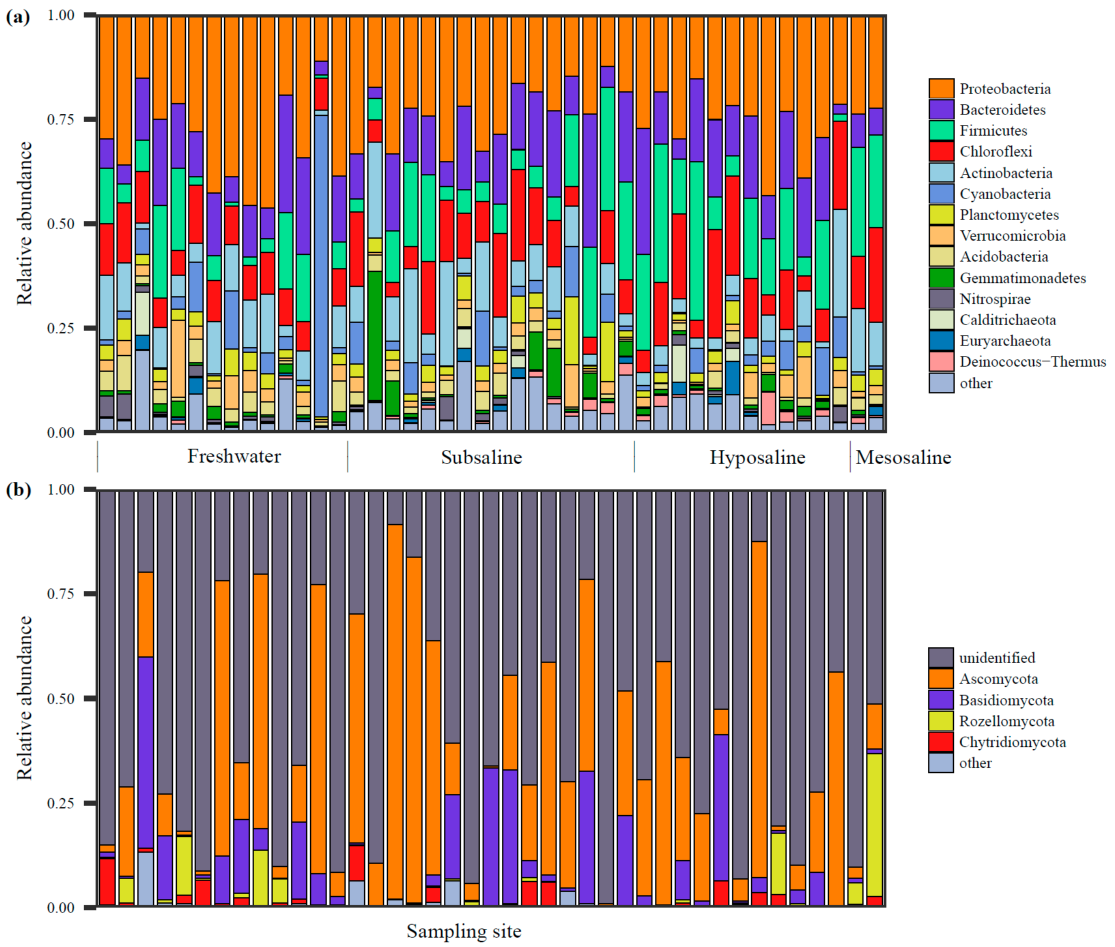


**Figure S5.** Stacked bars showing the bacterial (a) and fungal (b) community compositions in phylum level. The x-axes show sampling sites with increasing salinity from left to right. The salinity levels are denoted as freshwater (salinity < 0.5‰), subsaline (salinity = 0.5‰ ~ 3‰), hyposaline (salinity = 3‰ ~ 20‰) and mesosaline (salinity > 20‰). "Other" contains phyla with less than 0.5% average of all sites.

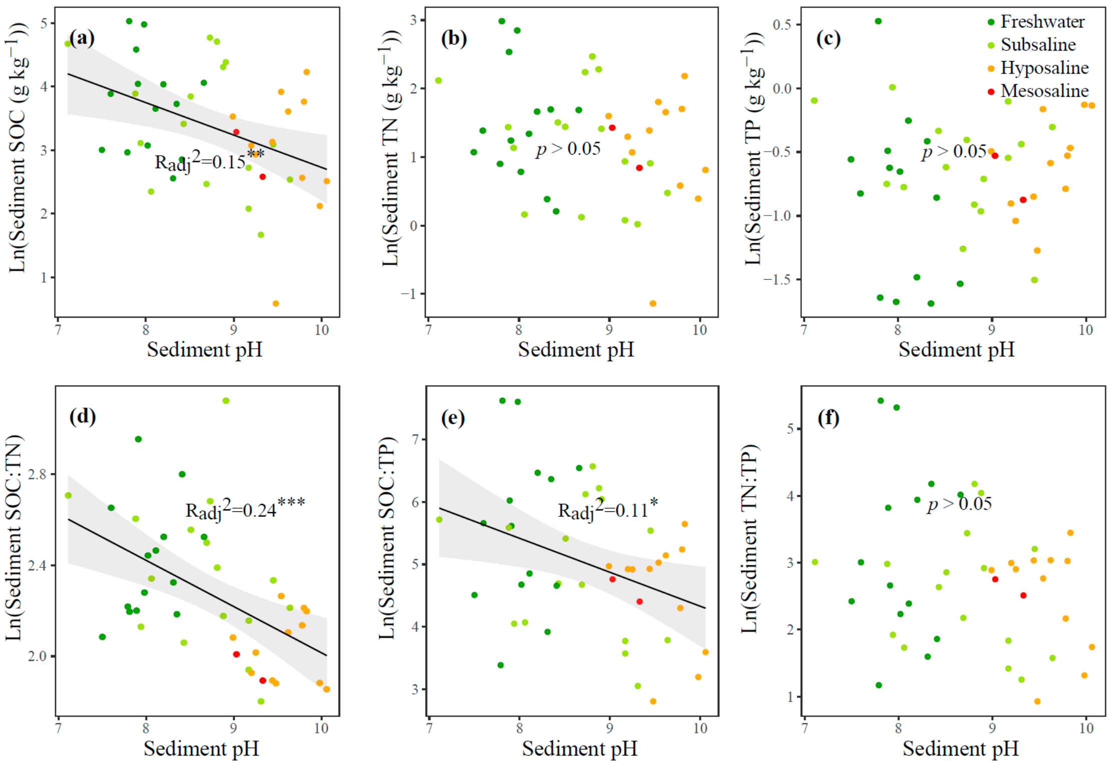


**Figure S6.** Linear regressions of sediment pH with the TOC, TN, TP, TOC:TN, TOC:TP and TN:TP and TP in the surface sediment (a-f). Only the significant fitted linear regressions are plotted with 95% confidence intervals filled in gray. The adjusted $R^2$ values of the linear models are denoted. * $p < 0.05$, ** $p < 0.01$, *** $p < 0.001$.

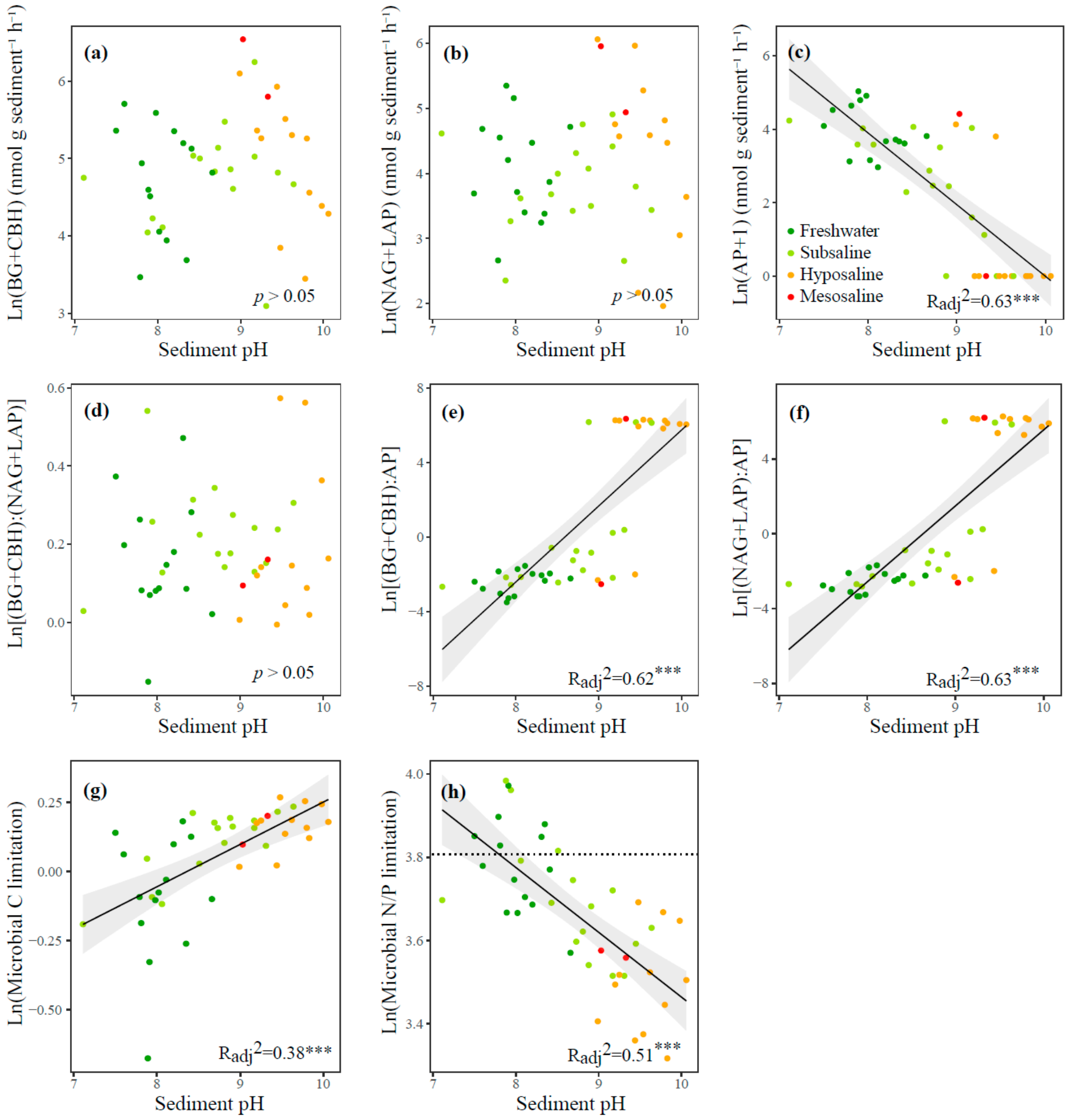


**Figure S7.** Linear regressions of sediment pH with enzyme activities involved in C-cycle (BG+CBH) (a), N-cycle (NAG+LAP) (b) and P-cycle (AP) (c), and with the ratio of C- to N-acquiring, C- to P-acquiring and N- to P-acquiring enzymatic activities (d-f), and with microbial C and N/P limitations (g-h). Y-axes in a-f are all log-transformed from original data to facilitate the fittings. Only the significant fitted linear regressions are plotted with 95% confidence intervals filled in gray. The adjusted $R^2$ values of the linear models are denoted. *** $p < 0.001$. BG, β-1,4-glucosidase; CBH, β-D-cellobiosidase; NAG, β-1,4-N-acetylglucosaminidase; LAP, L-leucine aminopeptidase; AP, alkaline phosphatase.

## References

1. Zhang W.; Liu Y.; Geng M.; Chen R.; Wang J.; Xue B., et al. Extracellular enzyme stoichiometry reveals carbon and nitrogen limitations closely linked to bacterial communities in China's largest saline lake. *Front Microbiol.* 2022, *13*, doi:10.3389/fmicb.2022.1002542.

2. Zhang W.; Chen R.; Meng F.; Yuan H.; Geng M.; Cheng L., et al. Ecosystem functioning is linked to microbial evenness and community composition along depth gradient in a semiarid lake. *Ecol. Indic.* 2021, *132*, 108314. doi:10.1016/j.ecolind.2021.108314.
